# Impact of symptoms on quality of life before and after diagnosis of coeliac disease: results from a UK population survey

**DOI:** 10.1186/1472-6963-10-105

**Published:** 2010-04-27

**Authors:** Alastair M Gray, Irene N Papanicolas

**Affiliations:** 1 Health Economics Research Centre, Department of Public Health, University of Oxford, Old Road Campus, Oxford, UK; 2 LSE Health, London School of Economics and Political Science, Houghton Street, London, UK

## Abstract

**Background:**

Coeliac disease is a common chronic autoimmune disorder. Underdiagnosis is common and the quality of life impact of symptoms may be severe. We report a study of symptom duration and quality of life before and after diagnosis in a representative sample of people with diagnosed coeliac disease in the UK.

**Methods:**

Postal questionnaire of 2000 people with diagnosed coeliac disease, requesting information on date of diagnosis, type and duration of symptoms, and quality of life before and after diagnosis using the EQ-5D instrument.

**Results:**

The survey response rate was 40% (788/2000). Mean duration of symptoms prior to diagnosis was 13.2 years, with some evidence of shorter duration in recent years. Respondents reported a mean of 13 consultations with their GP about their symptoms prior to diagnosis. The mean utility value of pre-diagnosis quality of life was 0.56, compared to 0.84 at time of survey, a highly statistically significant improvement of 0.27 (95% c.i. 0.25, 0.30).

**Conclusions:**

The symptoms of undiagnosed coeliac disease are associated with a prolonged and substantial decrement to quality of life. These results strengthen the case for detailed examination of the cost-effectiveness of improved methods of detection and diagnosis, including population screening.

## Background

Coeliac disease is a common chronic autoimmune disorder with a prevalence amongst adults and children approaching 1% of the population in international studies [1,2]. Underdiagnosis is common [3] and some studies have reported frequent presentation with symptoms over many years prior to diagnosis [4], although rates of diagnosis are increasing in many countries [5]. Once diagnosed, adherence to treatment involving the lifelong elimination of wheat, rye and barley from the diet results in significant clinical improvement for most patients. A small number of studies have examined the quality of life of coeliac patients, but these have typically focussed on the quality of life of patients after diagnosis in relation to the general population, and in particular on the impact of a gluten free diet, and have relied on small samples and instruments that do not facilitate comparison [6-8]. Here we report on a survey of people with coeliac disease, in which information was collected on the duration and types of symptoms experienced prior to diagnosis, the number of consultations about these symptoms prior to diagnosis, and quality of life before and after diagnosis using for the first time in this population the EQ-5D instrument, a generic utility-based instrument that is widely used in surveys and favoured in technology assessment as it facilitates comparison across disease areas and the general population [9,10]. We focus on comparisons before and after diagnosis, but also examine whether the introduction of serological testing, approximately between 1993 and 2000 [5], altered characteristics at diagnosis.

## Methods

A representative sample of 2,000 individuals was drawn from the membership list of Coeliac UK, the leading charity working for people with coeliac disease and dermatitis herpetiformis (DH) in the UK with a total membership of 70,000 or 56% of the estimated 125,000 people with a clinical diagnosis of coeliac disease in the UK as of October 2007. A short questionnaire was designed, containing questions on demographic characteristics, time since diagnosis, type and duration of symptoms prior to diagnosis, and quality of life before and after diagnosis, using the EQ-5D questionnaire. Other data collected in the questionnaire, for example on impact on daily activities and out-of-pocket costs associated with coeliac disease before and after diagnosis, are not reported here. The symptom lists were devised based on existing literature, and revised following piloting of the questionnaire with a local Coeliac UK members group. A copy of the questionnaire accompanies this article [Additional file 1].

Where members were known to be under 18 years of age, the parent or guardian was asked to complete the questionnaire on behalf of or with the member. The questionnaire was publicised in the Coeliac UK Newsletter sent to all members, and mailed with a covering letter and prepaid return envelope to the sample. The sample approached was stratified by each country of the UK, postal town or county, and date of joining Coeliac UK. Reminders to return the questionnaire were also publicised in Coeliac UK newsletters. The survey was conducted in 2007.

Data were entered onto a database, and a 10% sample was double-entered to check whether any coding inconsistencies were concentrated in particular parts of the questionnaire. These were then resolved by discussion and re-examination of data.

No imputation was conducted to deal with missing data, and results are presented using complete cases for the relevant question or combination of questions. T-tests were performed to assess whether missing cases were significantly different from compete cases.

Responses to the EQ-5D questions were given a quality of life valuation using the UK population tariff [11], and were compared with population norms derived from a national survey [12], having age- and sex-standardised to the survey population. Proportions reporting no problems before diagnosis and now in response to each EQ-5D question were compared using McNemar's chi-squared test. Results were analysed in SPSS version 15.^©^

### Data

788 of 2000 questionnaires were returned, a response rate of just under 40%. The age and sex distribution of respondents were compared with the Coeliac UK general membership and no statistically significant differences were observed. The level of non-response varied from 2/788 (<0.5%) for presence of symptoms to 133/788 (17%) for number of GP consultations prior to diagnosis.

## Results

93% of respondents (728/783) were the only member of their household with coeliac disease, with 7% sharing their household with at least one other person diagnosed with coeliac disease.

28% of respondents (220/777) were male and 72% (557/777) female. The mean age (SD) of respondents was 52 (18) years, with a range from 2 to 89. 12% (97/777) of respondents were aged less than 18 years, and 10% (80/777) were aged 65 or over. On average respondents were aged 41.3 years (SD 19) when they were diagnosed with CD. The mean age at diagnosis was 39 (SD 18) for those diagnosed before the year 2000, compared to 44 for those diagnosed after 2000, a significant difference of 5 years (95% ci 2.7 to 8.0).

Table 1 reports the frequency and duration of reported symptoms prior to diagnosis. The most common symptoms were abdominal pain/bloating (71%), diarrhoea (70%), anaemia (65%), chronic fatigue (62%), and weight loss (61%). 78% of respondents (605/777) reported at least 4 symptoms, and only 1% (6/777) of respondents reported no symptoms prior to diagnosis.

**Table 1:** Frequency and duration of symptoms prior to diagnosis of coeliac disease.

Symptom:	Number (%) reporting symptoms (of 777 respondents)			Duration of symptoms (years)	
	**Number**	**%**	**(95% CI)**	**Mean**	**(95% CI)**
	
Any symptom	771	99	(98, 100)	13.2	(12.1, 14.4)
Abdominal pain/bloating	556	71	(67, 74)	7.9	(7.0, 8.7)
Diarrhoea	553	70	(67, 73)	6.9	(6.1, 7.8)
Anaemia	509	65	(61, 680	11.5	(10.5, 12.6)
Chronic fatigue	488	62	(59, 650	7.1	(6.3, 7.9)
Weight loss	479	61	(57, 640	5.5	(4.7, 6.2)
Flatulence	368	47	(43, 50)	9.5	(8.6, 10.4)
Mouth ulcer	236	30	(27, 33)	11.2	(10.1, 12.3)
Headache	232	30	(26, 33)	10.3	(9.3, 11.3)
Joint pain	220	28	(25, 31)	8.2	(7.4, 9.0)
Skin rash	208	26	(23, 30)	9.9	(8.9, 10.9)
Constipation	207	26	(23, 29)	12.6	(11.5, 13.6)
Depression	185	24	(21, 27)	9.2	(8.3, 10.1)
Other symptoms	163	21	(18, 24)	5.4	(4.8, 6.1)
Osteoporosis	91	12	(10, 14)	7.7	(6.7, 8.6)
Ataxia	39	5	(3, 7)	6.1	(5.2, 6.9)
No symptoms	6	1	(0, 2)		

The mean duration of specific symptoms ranged from 12.6 years for constipation to 5.5 years for weight loss. The average duration of any symptom before diagnosis across the whole sample was 13.2 (SD 16.0) years. The duration of symptoms in those diagnosed before and after the year 2000 was 14.5 and 12.0 years respectively, a significant difference of 2.5 years (95% CI 0.2. 4.9).

655 respondents (83%) provided information about the number of times they had seen their GP about their symptoms during the period prior to diagnosis, and Table 2 reports details. On average, respondents consulted their GP 13.0 times about their symptoms. This was related to duration of symptoms: those whose symptoms had lasted for less than 1 year before diagnosis consulted GPs on average 4.5 times for symptom related consultations, whereas those whose symptoms had lasted for 20 or more years had consulted their GP on average 27.7 times for symptom related consultations. The average number of pre-diagnosis visits to GPs about symptoms was 7.0 amongst those diagnosed after the year 2000 compared to 17.4 amongst those diagnosed before 2000, a mean difference of 10.4 visits (95% CI 2.3, 18.5). Adjusted for age of respondent and duration of symptoms, this difference remained a statistically significant 11.4 visits (95% CI 1.9, 20.8).

**Table 2:** Mean number of GP consultations pre-diagnosis about symptoms, by duration of symptoms.

	Number of consultations		
Duration of all symptoms in years:	**Mean**	**N**	**95% CI**
>1	4.5	106	(3.5, 5.5)
1-5	9.2	170	(4.1, 14.3)
5-10	8.4	79	(6.1, 10.7)
10-20	8.9	95	(6.5, 11.4)
>20	27.7	155	(11.4, 44.1)
Total	13.0	605	(8.5, 17.5)

Table 3 reports the number and percent of respondents at different levels of each of the 5 questions comprising the EQ-5D, before and after diagnosis, and the corresponding percentages at each level in the general population of England. Results are shown for the 697 of 788 respondents (88%) completing EQ-5D questionnaires for both their pre-diagnosis and their current health state.

**Table 3:** Respondents' self-reported health on EQ-5D before diagnosis of coeliac disease (retrospective) and now: number and percent by level of response, and UK population norms (percent)*^1^.

EQ-5D question:	Level 1: No problems		Level 2: Some problems		Level 3: Severe problems	
	**N**	**%**	**N**	**%**	**N**	**%**
	
**Mobility:**						
before diagnosis	524	75	159	23	14	2
Now	602	86*	93	13	2	0
*UK population norm*		*78*		*22*		*0*
**Self-care:**						
before diagnosis	648	93	35	5	14	2
Now	673	97*	18	3	6	1
*UK population norm*		*93*		*6*		*0*
**Usual activities:**						
before diagnosis	403	58	249	36	45	6
Now	574	82*	116	17	7	1
*UK population norm*		*78*		*19*		*3*
**Pain:**						
before diagnosis	153	22	349	50	195	28
Now	416	60*	252	36	29	4
*UK population norm*		*58*		*37*		*4*
**Anxiety/depression:**						
before diagnosis	351	50	259	37	87	12
Now	518	74*	163	23	16	2
*UK population norm*		*75*		*23*		*2*

*Proportion reporting no problem significantly different now compared to before diagnosis (McNemar-Bowker Chi-square test, p < 0.01)

^1 ^UK population norms from Health Survey for England 1996, standardised to age distribution of Coeliac UK survey respondents now.

In all five dimensions of the EQ-5D, the proportion of respondents reporting no problems was significantly higher at the time of the survey compared to before diagnosis: this was particularly pronounced in the pain dimension, with 60% reporting themselves to have no problems at the time of the survey, compared to only 22% prior to diagnosis of coeliac disease. The distribution of responses from a large general population survey in England, age- and sex-standardised to the survey respondent population, is also shown in Table 3: across all five health dimensions the proportion of respondents reporting no problems before diagnosis was lower than in the general population, but at time of survey was similar to or higher than in the general population.

Placing valuations on these EQ-5D health states using the British "tariff"^11 ^(Table 4), the mean quality of life before diagnosis was 0.56, (where 0 = death and 1 = full health), and 0.84 at the time of the survey, indicating a highly statistically significant improvement of 0.27 (95% c.i. 0.25, 0.30). By comparison, the average quality of life in the general population has been reported as 0.82 when age-sex-standardised to age of respondents at time of survey response, or 0.85 when age-standardised to age of respondents at age at diagnosis [12].

**Table 4:** Respondents' self-reported health on EQ-5D before diagnosis of coeliac disease (retrospective) and now: mean score on Visual Analogue Scale and mean tariff-based valuation of health state, and UK population norm^1^.

	Mean	95% CI
EQ-5D tariff:		
pre-diagnosis	0.56	(0.54, 0.59)
time of survey	0.84	(0.82, 0.85)
change	0.27	(0.25, 0.30)
*UK population norm, standardised to age distribution of respondents at:*		
*time of survey*	*0.82*	*(0.81, 0.83)*
*time of diagnosis*	*0.85*	*(0.84, 0.86)*
		
Visual Analogue Scale:		
pre-diagnosis	47%	(45, 49)
time of survey	79%	(78, 80)
change	32%	(30, 35)

^1 ^UK population norms from Health Survey for England 1996, standardised to age distribution of Coeliac UK survey respondents now.

On the Visual Analogue Scale, respondents rated their health at 47% before diagnosis (0 = worst imaginable state, 100 = best imaginable state), and at 79% now, a highly significant improvement of 32 (95% C.I. 30, 35) percentage points.

There was no clear evidence that the levels of health reported by respondents before and after diagnosis was related to duration of symptoms. Respondents who were diagnosed prior to 2000 had a mean EQ-5D tariff of 0.55, compared with 0.58 amongst those diagnosed after 2000, a small but not statistically significant difference of 0.03 (95% C.I. -0.02, 0.08). Similarly, the VAS was slightly but not significantly higher at diagnosis amongst those diagnosed after 2000 (49%) compared with those diagnosed before 200 (46%) (mean difference 3, 95% C.I. -1, 6).

**Figure 1:**
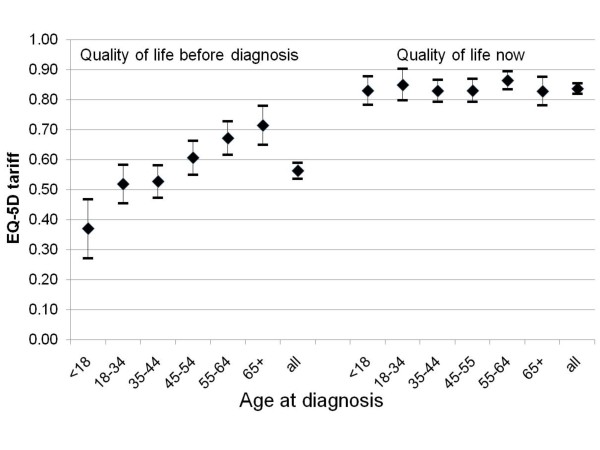
**Respondents' self-reported health on EQ-5D before diagnosis of coeliac disease (retrospective) and now: mean tariff-based valuation of health state, by age at diagnosis**.

Figure 1 shows levels of health reported by respondents, by age group, before diagnosis and at the time of the survey. Levels of health were clearly related to age before diagnosis, rising from 0.52 (95% C.I. 0.45, 0.58) amongst those aged 18-34 when diagnosed to 0.71 (95% C.I. 0.65, 0.78) amongst those aged 65 and over at diagnosis. No such differences between age groups were evident in reported quality of life at time of survey. However, all age groups reported a significant improvement in quality of life at the time of the survey compared to the period prior to diagnosis.

## Discussion

In this study we have shown that the quality of life of people with undiagnosed symptomatic coeliac disease is substantially reduced compared to the general population, and increases markedly after diagnosis. The difference (0.56 prior to diagnosis (where 0 = death and 1 = full health) to 0.84 at the time of the survey), is quantitatively similar to the quality of life impact of severe events such as stroke [13].

Our study found a mean duration of symptoms of 13.2 years; although there was some evidence that this had fallen since the widespread adoption of serological testing in the 1990s, the mean duration of symptoms of those diagnosed after the year 2000 was still 12 years. These durations are similar to the 11 years with symptoms prior to diagnosis reported in a large American study, which also showed some evidence of reduced duration of symptoms following the introduction of serological testing [14].

Previous studies of quality of life associated with coeliac disease have not focused on differences before and after diagnosis, and have not used the EQ-5D. One advantage of the EQ-5D is that it is a widely used generic quality of life instrument that permits comparison across many different disease areas, and is therefore particularly useful in assessing the comparative cost-effectiveness of a wide range of different interventions: for this reason the EQ-5D is the only quality of life instrument specifically recommended for calculating quality adjusted life years by the National Institute of Health and Clinical Excellence in its recommended methods for technology appraisal [10]. The EQ-5D derived estimates of utility before and after diagnosis reported here, and the estimated duration of symptoms prior to diagnosis, may be helpful in assessing the cost-effectiveness of improved methods of screening and detection; such studies to date have typically concentrated on cost per case detected [15], often in population sub-groups [16] or have specifically excluded quality of life due to the prior lack of reliable estimates [17]. A systematic review undertaken to inform the cost-effectiveness modelling that formed part of the NICE Guidelines on celiac disease published in 2009 was unable to find any utility estimates in the literature at that time [18].

Our estimates of quality of life prior to diagnosis of coeliac disease are based on retrospective assessment; such methods are unavoidable in the absence of very large long-term prospective studies, and have been used before with different instruments and in different disease areas [19,20], but it is not known whether the results obtained would be comparable with those derived from prospective studies, and there is a lack of information on this with the EQ-5D or indeed other instruments. Similarly, although there is no evidence that the respondents to this survey were different to non-respondents, it is possible that the population from which the sample was drawn - members of Coeliac UK, stratified by area and duration of membership - are in some way unrepresentative of the entire population of those with diagnosed coeliac disease. However, given the magnitude of the quality of life differences reported in this study, and given that the charity has 56% of all diagnosed patients enrolled in its membership, it seems unlikely that any recall or sampling bias could seriously alter the results.

## Conclusions

The symptoms of undiagnosed coeliac disease are associated with a prolonged and substantial decrement to quality of life. In light of these results, the case for detailed examination of the cost-effectiveness of improved methods of detection and diagnosis, including population screening, seems compelling.

## Competing interests

The authors declare that they have no competing interests.

## Authors' contributions

AMG designed the study, participated in the data analysis and drafted the manuscript. ING participated in the design of the survey questionnaire, transcribed, coded and analysed the responses and helped to draft the manuscript. Both authors read and approved the final manuscript.

## Pre-publication history

The pre-publication history for this paper can be accessed here:

http://www.biomedcentral.com/1472-6963/10/105/prepub

## Supplementary Material

Additional file 1 **A copy of the questionnaire used for the survey.** This is a nine-page questionnaire in PDF format. Some sections relate the quality of life survey reported here, and some to other data collected. Click here for file
