# Supplementary material for: Impact of symptoms on quality of life before and after diagnosis of coeliac disease: results from a UK population survey
Source: BMC Health Serv Res. 2010 Apr 27;10:105. doi: 10.1186/1472-6963-10-105 (PMC2907763; doi:10.1186/1472-6963-10-105)

# The Impact of Coeliac Disease on Your Life: A Survey of Your Views

University of Oxford
(Health Economics Research Centre)
&
Coeliac UK

Questionnaire – September 2006

Please fill out the following questionnaire and return it to us in the stamped addressed envelope provided. Thank you for all your help.

## Section A: General Respondent Details

**1.** Are you Female ☐ Male ☐

**2.** What is your age (in years)?

☐

**3.** What was your age (in years) when you were diagnosed with coeliac disease?

☐

**4.** Please state the number of people living in your household (not including yourself).

☐

**5.** How many of the members of your household have been diagnosed with coeliac disease (not including yourself)?

☐

**6.** Did you have any of the following symptoms prior to diagnosis? For each symptom that you had, please tick the box next to it and indicate the **approximate** time that you had it for. If you had no symptoms please tick the 'none of the above' box.

| Symptom | Tick if Experienced | Approximate Time Experienced | |
|---|---|---|---|
| | | Months | Years |
| Diarrhoea | ☐ | | |
| Constipation | ☐ | | |
| Fatigue | ☐ | | |
| Abdominal Pain | ☐ | | |
| Flatulence | ☐ | | |
| Dyspepsia | ☐ | | |
| Headaches | ☐ | | |
| Skin Rashes | ☐ | | |
| Mouth Ulcers | ☐ | | |
| Mood Swings | ☐ | | |
| Depression | ☐ | | |
| Weight Loss | ☐ | | |
| Loss of muscular coordination (Ataxia) | ☐ | | |
| Other, please specify: ____________________ | ☐ | | |
| None of the above | ☐ | | |

## End of Section A.

# Section B: Out-of-Pocket Costs Associated with Coeliac Disease

## Section B1: Costs Prior to Diagnosis

The following section asks about your out-of-pocket costs when you had symptoms related to coeliac disease but had **not yet been diagnosed.** Please answer all questions for the **whole** of this time period. We realise that it may be difficult to give precise answers but it would be very useful to our study if you could give us your **best estimate**.

**7.** During the time that you had symptoms related to coeliac disease but had not yet been diagnosed, how many times did you see your GP **about your symptoms**?

**8.** During the time that you had symptoms related to coeliac disease but had not yet been diagnosed, approximately how many days did you take off work **due to your symptoms?**

**9.** During the time that you had symptoms related to coeliac disease but had not yet been diagnosed, approximately how much did you spend (*including all direct costs and any travel expenses*), on the following (if unsure of precise costs, please give us your **best estimates**):

(a). Private consultation(s) with gastroenterologists and/or other medical doctors about your symptoms:

(b). Private consultation(s) with nutritional therapists / dietitians / homeopaths / and/or other therapists about your symptoms:

(c). Private allergy testing *(e.g. the York Test*):

(d). Over-the-counter medications to alleviate symptoms *(e.g. immodium plus, dioralite bonjella, gaviscon)*:

(e). Dietary products to help experiment with different diets to alleviate symptoms *(e.g. wheat-free diet, dairy-free diet)*:

(f). Food supplements *(e.g. vitamins)*:

(g). Books, DVDs, or videos on symptoms / symptom relief methods:

(h). Other, please give a brief description ____________________

**Section B2: Costs Following Diagnosis**

The following section will ask you about your out-of-pocket costs in the time **after you were diagnosed** with coeliac disease. Please note that these questions will ask you for **average yearly costs** since diagnosis. We realise that it may be difficult to give precise answers for the following questions, but it would be very useful to our study if you could give us your **best estimates**.

**10.** Since diagnosis, how many times per year, on average, do you see your GP **about your coeliac disease or related symptoms?**

**11.** Since diagnosis, approximately how many days per year, on average, have you taken off work due to **your coeliac disease or related symptoms?**

**12.** Have you had to change your work as a result of your diagnosis with coeliac disease?

Yes ☐ No ☐

**13.** Since diagnosis, approximately how much have you spent, **on average per year** on the following (if unsure of precise costs, please give us your **best estimates**):

(a). Private consultation(s) with gastroenterologists and/or other medical doctors about your coeliac disease or related symptoms *(please include fees, as well as travel and any other expenses)*:

[    ]

(b). Private consultation(s) with nutritional therapists / dietitians / homeopaths / and/or other therapists about your coeliac disease or related symptoms *(please include fees, as well as travel and any other expenses)*:

[    ]

(c). Over-the-counter medications to alleviate symptoms *(e.g. immodium plus, dioralite, bonjella, gaviscon)*:

[    ]

(d). Food supplements *(e.g. vitamins)*:

[    ]

(e). Books, DVDs or videos on symptoms / symptom relief methods:

[    ]

(f). Other, please give a brief description____________________

[    ]

**14.** Do you get any gluten-free food on prescription?

Yes ☐ No *(If no, please go directly to Q17)* ☐

**15.** If yes, approximately how many prescription items per month do you receive? (1 item = 1 packet of gluten-free food stuff)

[    ]

**16.** How are your prescription items paid for:

Through a prescription pre-payment certificate/season ticket ☐

Through a prescription charge per item ☐

You are exempt from charges ☐

**17.** Apart from prescription foods, could you please indicate by **approximately** how much your weekly household shopping bill for food (for the whole household) has changed since you were diagnosed with coeliac disease? (please tick the box that applies)

Has increased by over £20 ☐
Has increased by £10-£20 ☐
Has increased by £0-£10 ☐
Has stayed the same ☐
Has decreased by £0-£10 ☐
Has decreased by £10-£20 ☐
Has decreased by over £20 ☐

**18.** Since diagnosis how have your eating patterns at work (lunch) changed? *(tick the answer that best applies)*

More likely to eat food prepared at work ☐
Less likely to eat food prepared at work ☐
No change ☐
Not applicable *(i.e. self employed)* ☐

**19.** Since diagnosis, by how much have the average **weekly** costs of eating lunch at work changed? *(Please tick the box corresponding to the choice that applies and indicate your* ***best estimate*** *of the amount by which they have changed).*

| Change | Tick Box | Approximate Cost (£) |
|---|---|---|
| They have increased | ☐ | |
| They have decreased | ☐ | |
| The have stayed the same | ☐ | n/a |

**20.** Since diagnosis are you more or less likely to eat a meal outside home (during evenings, on weekends and on holiday)?

More likely ☐
Less likely ☐
The same ☐

**21.** Since diagnosis when you do eat a meal outside home (during evenings, on weekends and on holiday), **how has your coeliac disease affected the average cost of a meal**? *(Please tick the box corresponding to the choice that applies and indicate your* ***best estimate*** *of the amount by which they have changed).*

| Change | Tick Box | Approximate Cost (£) |
|---|---|---|
| The average cost per meal has increased | ☐ | |
| The average cost per meal has decreased | ☐ | |
| The average cost per meal has not been affected | ☐ | n/a |

**22.** Since diagnosis are you more or less likely to go on holiday?

More likely ☐

Less likely ☐

The same ☐

**23.** Have you incurred any of the following household costs at any time since diagnosis? Please tick all that apply and indicate their approximate cost.

| Household Cost | Tick if Applies | Approximate Cost (£) |
|---|---|---|
| Buy new toaster(s) | ☐ | |
| Buy bread maker | ☐ | |
| Buy new chopping boards | ☐ | |
| Buy new cooking utensils | ☐ | |
| Buy new cooking books | ☐ | |
| Buy new food storage containers<br>*(e.g. tupperwear, food bags)* | ☐ | |
| Other, please specify<br>________________________________ | ☐ | |

**End of Section B.**

## Section C: Quality of Life

Please indicate which statements best describe your health state prior to diagnosis by placing a tick in **one** of the three options for each question in column 1. Then repeat this process for your current health state by placing a tick in **one** of the three options for each question in column 2.

| **Health State** | **Column 1: Before Diagnosis** | **Column2: Current State** |
|---|---|---|
| **Question 1: Mobility** | | |
| I had/have no problems in walking about | ☐ | ☐ |
| I had/have some problems in walking about | ☐ | ☐ |
| I was/am confined to bed | ☐ | ☐ |
| **Question 2: Self-care** | | |
| I had/have no problems with self care | ☐ | ☐ |
| I had/have some problems with washing or dressing myself | ☐ | ☐ |
| I was/am unable to wash and dress myself | ☐ | ☐ |
| **Question 3: Usual activities** | | |
| I had/have no problem in performing my usual activities *(e.g. work, study, housework, leisure activity)* | ☐ | ☐ |
| I had/have some problems in performing my usual activities | ☐ | ☐ |
| I was/am unable to perform my usual activities | ☐ | ☐ |
| **Question 4: Pain/Discomfort** | | |
| I had/have no pain or discomfort | ☐ | ☐ |
| I had/have moderate pain or discomfort | ☐ | ☐ |
| I had/have extreme pain or discomfort | ☐ | ☐ |
| **Question 5: Anxiety/Depression** | | |
| I was/am not anxious or depressed | ☐ | ☐ |
| I was/am moderately anxious or depressed | ☐ | ☐ |
| I was/am extremely anxious or depressed | ☐ | ☐ |

In order to measure how good or bad a health state is we have drawn two scales from 1 to 100. The best health state you can imagine is marked by 100 and the worst health state is marked by 0.

We would like you to indicate on Scale 1 how good or bad your own health was, in your opinion, **prior to your diagnosis**. Please do this by drawing a **single line** from box 1 to whichever point on Scale 1 indicates how your health state was.

We would then like you to indicate on the Scale 2 how good or bad your own health is, in your opinion, **currently**. Please do this by drawing a **single line** from box 2 to whichever point on Scale 2 indicates how your health state is.

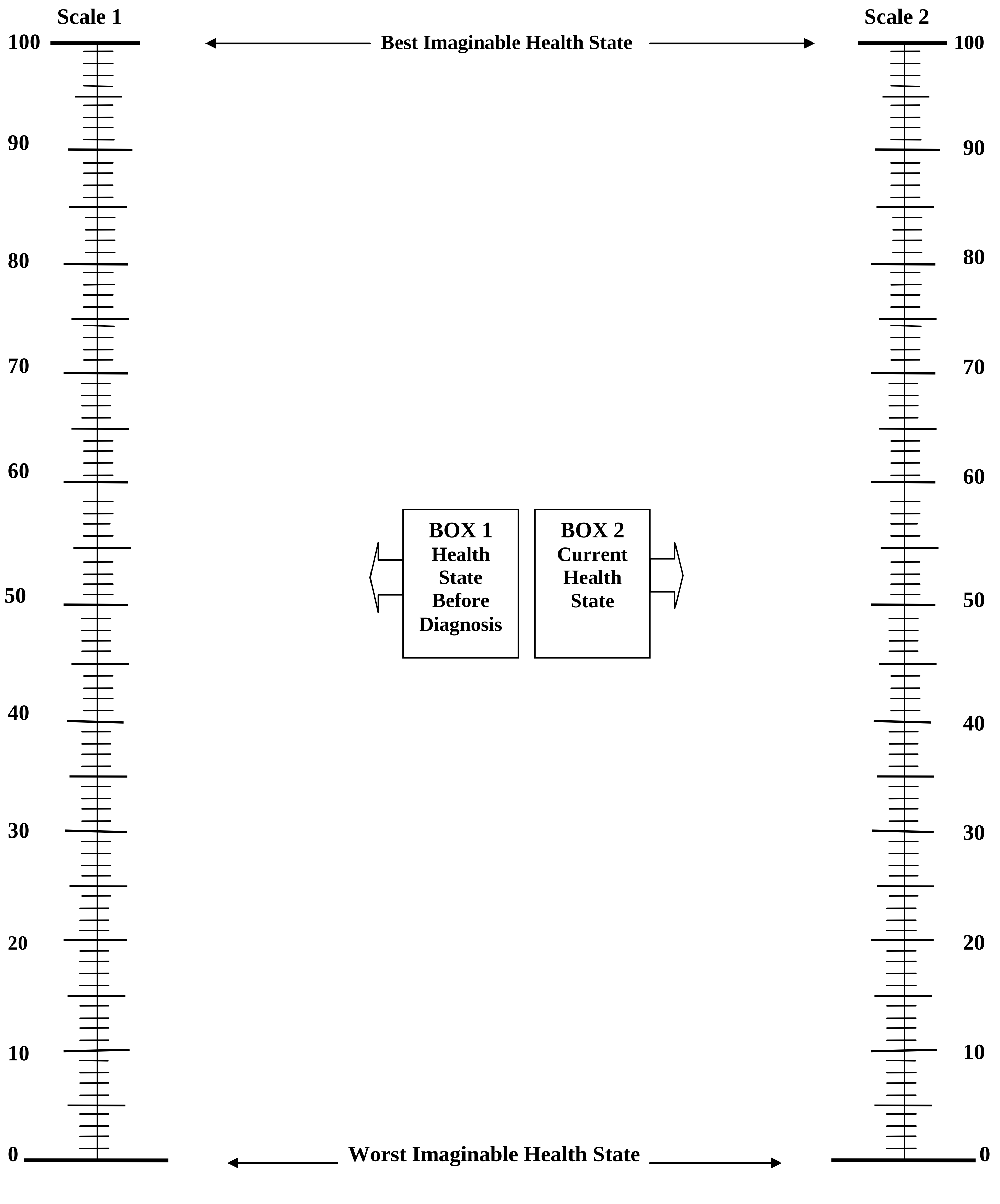

Supplement: Additional file 1 — A copy of the questionnaire used for the survey. This is a nine-page questionnaire in PDF format. Some sections relate the quality of life survey reported here, and some to other data collected. [file 1472-6963-10-105-S1.PDF]
